# Core charge distribution and self assembly of columnar phases: the case of triphenylenes and azatriphenylenes

**DOI:** 10.1186/1752-153X-1-15

**Published:** 2007-06-08

**Authors:** Silvia Orlandi, Luca Muccioli, Matteo Ricci, Roberto Berardi, Claudio Zannoni

**Affiliations:** 1 Dipartimento di Chimica Fisica ed Inorganica and INSTM, Università di Bologna, Viale del Risorgimento, 4, I-40136 Bologna, Italy

## Abstract

**Background:**

The relation betweeen the structure of discotic molecules and columnar properties, a crucial point for the realization of new advanced materials, is still largely unknown. A paradigmatic case is that hexa-alkyl-thio substituted triphenylenes present mesogenic behavior while the corresponding azatriphenylenes, similar in shape and chemical structure, but with a different core charge distribution, do not form any liquid crystalline mesophase. This study is aimed at investigating, with the help of computer simulations techniques, the effects on phase behaviour of changes of the charge distribution in the discotic core.

**Results:**

We described the shape and the pair, dispersive and electrostatic, interactions of hexa alkyl triphenylenes by uniaxial Gay-Berne discs with embedded point charges. Gay-Berne parameters were deduced by fitting the dispersive energies obtained from an atomistic molecular dynamics simulation of a small sample of hexa-octyl-thio triphenylene molecules in columnar phase, while a genetic algorithm was used to get a minimal set of point charges that properly reproduces the ab anitio electrostatic potential. We performed Monte Carlo simulations of three molecular models: the pure Gay-Berne disc, used as a reference, the Gay-Berne disc with hexa-thio triphenylene point charges, the Gay-Berne disc with hexa-thio azatriphenylene point charges. The phase diagram of the pure model evidences a rich polymorphism, with isotropic, columnar and crystalline phases at low pressure, and the appearance of nematic phase at higher pressure.

**Conclusion:**

We found that the intermolecular electrostatic potential among the cores is fundamental in sta-bilizing/destabilizing columnar phases; in particular the triphenylene charge distribution stabilizes the columnar structure, while the azatriphenylene distribution suppresses its formation in favor of the nematic phase. We believe the present model could be successfully employed as the basis for coarse-grained level simulations of a wider class of triphenylene derivatives.

## Background

**Figure 1:**
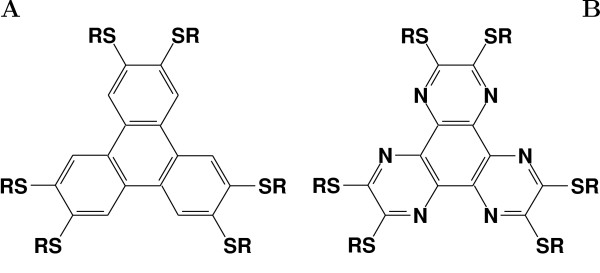
**Chemical structures**. Chemical structure of hexa-alkyl-thio triphenylene (A: R = H ⇒ HT-T, R = octyl ⇒ 8HT-T) and hexa-alkyl-thio azatriphenylene (B: R = H ⇒ HT-AT, R = octyl ⇒ 8HT-AT).

A central problem in the preparation of new advanced materials, and particularly those based on complex molecules such as liquid crystals, is understanding the relation between molecular structure and material properties. For discotics molecules spontaneously forming columnar materials, this relation is still largely unknown. Even if it is empirically clear that a suitable combination of a flat, aromatic core, and a number of laterally attached alkyl chains may result in spontaneous mesomorphic behaviour, a large body of observations remains unexplained. For instance, it is found that very few nematic discotics exist, differently from the case of elongated molecules [1,2], and that the spontaneous ordering transition upon cooling brings directly from an isotropic to a columnar phase. Moreover, given cores of similar size, why do some mesogens form well ordered columns much better than others? A paradigmatic example is that hexa-alkyl substituted triphenylenes present mesogenic behavior [1,3,4], while the corresponding azatriphenylenes, notwithstanding the apparent similarity in shape and chemical structure with the former compounds (see Figure 1), do not form any liquid crystalline mesophase [5] if not enforced by adding hydrogen bonding side groups [6,7]. This study is aimed at investigating the origin of this fact and more generally at examining, with the help of computer simulation techniques, the effects on phase behaviour of changes of the charge distribution in the discotic core.

Given their capability to provide microscopic details that cannot or are difficult to access with experimental techniques, computer simulations have already demonstrated their usefulness in the field of liquid crystals [8,9], even if the majority of studies has been carried out on calamitic-type mesogens. As liquid crystalline properties depend on collective molecular organization, the model has to contain on one hand enough atomic details to be chemically meaningful [10,11] while on the other, still be manageable in the simulation of large enough samples of different phases [12]. When the latter aspect is predominant, the choice of simplified models which describe the molecule with a single or a few interacting sites is to be preferred. In this specific niche, simulations based on the attractive-repulsive Gay-Berne (GB) potential [13] have been very successful in reproducing and even predicting a variety of liquid crystalline phases [14,15].

So far, however, GB simulations of discotics have not considered the real dimensions of the typical triphenylenic molecules giving columnar phases, and the aspect ratio of the actual mesogenic molecules (e.g. the core thickness/diameter ratio) has been typically overestimated. For instance, most studies [16-21], based on the parameterization of Emerson *et al*. [16] have used an aspect ratio of 0.345, taking into account only the dimensions of the rigid molecular core, disregarding the presence of alkyl chains, which actually play a crucial role in determining the mesogenic properties of these compounds. X-ray experimental studies have demonstrated [3] that a more meaningful aspect ratio, estimated as the ratio between the cofacial and the intercolumnar distances in non-tilted phases, is hardly higher than 0.2 for triphenylenes, and in some cases, like hexabenzocoronene derivatives, even lower [22]. Despite this, until now only one simulation work, by Ryckaert and coworkers, reports the phase diagram of GB discs with lower aspect ratio [20].

Considering the necessity of reparameterizing the GB potential for triphenylenes, we attempted the investigation of the differences between hexa-thio triphenylene and hexa-thio azatriphenylene in two separate modelling/simulation steps. In the first we derived and parameterized a new GB molecular model for triphenylene-based compounds, by fitting the dispersive energies and the pair distributions calculated from the trajectories of an atomistic molecular dynamics (MD) simulation of chargeless hexa-octyl-thio triph-enylene (8HT-T). Then, by employing extensive Monte Carlo (MC) simulations we obtained the pressure-temperature phase diagram of the model, which shows crystalline, columnar, nematic, isotropic and gas regions. In the second step, we calculated, via *ab initio *methods, the electrostatic potential of hexa-thio triphenylene (HT-T) and hexa-thio azatriphenylene (HT-AT) cores and approximated them with two minimal sets of point charges [23]. We have studied, at a selected pressure, the variations induced on the phase behaviour of the two molecules by adding to the GB potential the point charges stemming from the HT-T and HT-AT electrostatic potential respectively.

## Results and Discussion

### Modelling

We modelled the total intermolecular potential as a sum of two additive terms: the first represents the dispersion forces and repulsive interactions between both the cores and the thioalkyl chains, and is intended to be a general model for a class of hexa-alkyl triphenylene derivatives with chain length ranging approximately from 5 to 9 atoms; the second is an electrostatic contribution, which is aimed at reproducing the Coulomb interactions between the molecular cores and which specifically depends on their chemical nature, here investigated for the triphenylene and azatriphenylene cases (Figure 1).

### Derivation of Gay-Berne parameters

The shape and the dispersive interactions of hexa-alkyl triphenylenes were described with an uniaxial repulsive-attractive GB [13] disc. The model considers only in an effective way the influence of molecular flexibility, by averaging over all the chain conformations.

This GB potential (equation 1) defines the interaction between two identical molecules *A *and *B *as a function of the intermolecular distance vector **r**_*AB *_and of the orientation of the molecular orientation vectors **u**_*A *_and **u**_*B*_, with the help of six parameters: the exponents *μ *and *ν *which allow a tuning of the orientational dependence of the energy; the contact distances *σ*_*ff *_(face-face) and *σ*_*ee *_(edge-edge), in unit *σ*_0_; the strength parameters *ε*_*ff *_and *ε*_*ee*_, in unit *ε*_0_. The parameters *σ*_*ff *_and *σ*_*ee *_are respectively the lower and upper limit of the contact distance function *σ*(**u**_*A*_, **u**_*B*_, **r**_*AB*_); the parameters *μ*,*ν*, *ε*_*ff *_and *ε*_*ee *_appear in the definition of the interaction function *ε*(**u**_*A*_, **u**_*B*_, **r**_*AB*_) [13].

UGB(uA,uB,rAB)=4ε0ε(uA,uB,rAB)×[(σffrAB−σ(uA,uB,rAB)+σff)12−(σffrAB−σ(uA,uB,rAB)+σff)6].
 MathType@MTEF@5@5@+=feaafiart1ev1aaatCvAUfKttLearuWrP9MDH5MBPbIqV92AaeXatLxBI9gBaebbnrfifHhDYfgasaacH8akY=wiFfYdH8Gipec8Eeeu0xXdbba9frFj0=OqFfea0dXdd9vqai=hGuQ8kuc9pgc9s8qqaq=dirpe0xb9q8qiLsFr0=vr0=vr0dc8meaabaqaciaacaGaaeqabaqabeGadaaakeaafaqaceGabaaabaGaemyvau1aaSbaaSqaaiabdEeahjabdkeacbqabaGccqGGOaakieqacqWF1bqDdaWgaaWcbaGaemyqaeeabeaakiabcYcaSiab=vha1naaBaaaleaacqWGcbGqaeqaaOGaeiilaWIae8NCai3aaSbaaSqaaiabdgeabjabdkeacbqabaGccqGGPaqkcqGH9aqpcqaI0aaniiGacqGF1oqzdaWgaaWcbaGaeGimaadabeaakiab+v7aLjabcIcaOiab=vha1naaBaaaleaacqWGbbqqaeqaaOGaeiilaWIae8xDau3aaSbaaSqaaiabdkeacbqabaGccqGGSaalcqWFYbGCdaWgaaWcbaGaemyqaeKaemOqaieabeaakiabcMcaPiabgEna0oaadeaabaWaaeWaaeaadaWcaaqaaiab+n8aZnaaBaaaleaacqWGMbGzcqWGMbGzaeqaaaGcbaGaemOCai3aaSbaaSqaaiabdgeabjabdkeacbqabaGccqGHsislcqGFdpWCcqGGOaakcqWF1bqDdaWgaaWcbaGaemyqaeeabeaakiabcYcaSiab=vha1naaBaaaleaacqWGcbGqaeqaaOGaeiilaWIae8NCai3aaSbaaSqaaiabdgeabjabdkeacbqabaGccqGGPaqkcqGHRaWkcqGFdpWCdaWgaaWcbaGaemOzayMaemOzaygabeaaaaaakiaawIcacaGLPaaadaahaaWcbeqaaiabigdaXiabikdaYaaaaOGaay5waaaabaWaamGaaeaacqGHsisldaqadaqaamaalaaabaGae43Wdm3aaSbaaSqaaiabdAgaMjabdAgaMbqabaaakeaacqWGYbGCdaWgaaWcbaGaemyqaeKaemOqaieabeaakiabgkHiTiab+n8aZjabcIcaOiab=vha1naaBaaaleaacqWGbbqqaeqaaOGaeiilaWIae8xDau3aaSbaaSqaaiabdkeacbqabaGccqGGSaalcqWFYbGCdaWgaaWcbaGaemyqaeKaemOqaieabeaakiabcMcaPiabgUcaRiab+n8aZnaaBaaaleaacqWGMbGzcqWGMbGzaeqaaaaaaOGaayjkaiaawMcaamaaCaaaleqabaGaeGOnaydaaaGccaGLDbaacqGGUaGlaaaaaa@964A@

In most GB simulations these parameters are simply assumed a priori, or typically varied to look for generic trends in the phase diagram [12]. Here we wish instead to use parameters which are consistent with a coarse grained approximation to specific molecular organisations obtained from an atomistic MD simulation of a small sample of 8HT-T molecules in columnar phases (see Experimental Section).

For each pair of molecules of the MD sample we calculated the Lennard-Jones intermolecular energy

ULJ=4∑i∈A∑j∈Bεij[(σijrij)12−(σijrij)6],
 MathType@MTEF@5@5@+=feaafiart1ev1aaatCvAUfKttLearuWrP9MDH5MBPbIqV92AaeXatLxBI9gBaebbnrfifHhDYfgasaacH8akY=wiFfYdH8Gipec8Eeeu0xXdbba9frFj0=OqFfea0dXdd9vqai=hGuQ8kuc9pgc9s8qqaq=dirpe0xb9q8qiLsFr0=vr0=vr0dc8meaabaqaciaacaGaaeqabaqabeGadaaakeaacqWGvbqvdaWgaaWcbaGaemitaWKaemOsaOeabeaakiabg2da9iabisda0maaqafabaWaaabuaeaaiiGacqWF1oqzdaWgaaWcbaGaemyAaKMaemOAaOgabeaaaeaacqWGQbGAcqGHiiIZcqWGcbGqaeqaniabggHiLdaaleaacqWGPbqAcqGHiiIZcqWGbbqqaeqaniabggHiLdGcdaWadaqaamaabmaabaWaaSaaaeaacqWFdpWCdaWgaaWcbaGaemyAaKMaemOAaOgabeaaaOqaaiabdkhaYnaaBaaaleaacqWGPbqAcqWGQbGAaeqaaaaaaOGaayjkaiaawMcaamaaCaaaleqabaGaeGymaeJaeGOmaidaaOGaeyOeI0YaaeWaaeaadaWcaaqaaiab=n8aZnaaBaaaleaacqWGPbqAcqWGQbGAaeqaaaGcbaGaemOCai3aaSbaaSqaaiabdMgaPjabdQgaQbqabaaaaaGccaGLOaGaayzkaaWaaWbaaSqabeaacqaI2aGnaaaakiaawUfacaGLDbaacqGGSaalaaa@5F47@

using the atomic coordinates stored from the MD simulation at intervals of 10 picoseconds. We computed the intermolecular vectors **r**_*AB *_of all pairs of molecules joining the centers of mass coordinates, and determined the two molecular orientations **u**_*A *_and **u**_*B *_as the eigenvectors of the molecular inertial frames corresponding to the maximum eigenvalues and calculated the corresponding GB energies. In order to obtain the best set of parameters for the system, we fitted all MD intermolecular potential energy points {**r**_*AB*_, **u**_*A*_, **u**_*B*_, U_*LJ*_} with the Gay-Berne expression by minimizing the total squared residuals ∑_*A,B*;*A*≠*B*_[*U*_*LJ*_(*A, B*) - *U*_*GB*_(*A, B*)]^2^. In the fitting procedure, the maximum GB energy was set to 10 kcal/mol and we also enforced the constraint of *ε*_*ff *_> *ε*_*ee*_. These two shape parameters were optimized using the Nelder-Mead symplex algorithm, while the *μ*, *ν*, *σ*_*ff*_, *σ*_*ee *_values were heuristically estimated by performing different series of minimization runs. The final parameters are: *μ *= 1, *ν *= 0, *σ*_*ee *_= 19.25 Å, *σ*_*ff *_= 3.75 Å, *ε*_*ee *_= 9 kcal/mol, and *ε*_*ff *_= 60 kcal/mol.

### Phase behaviour of charge-less model for triphenylene

Once the GB potential was parametrized, we studied by computer simulations the phase diagram of this coarse-grained molecular model (without charges: system Q0), by performing both MC cooling and heating scans.

**Figure 2:**
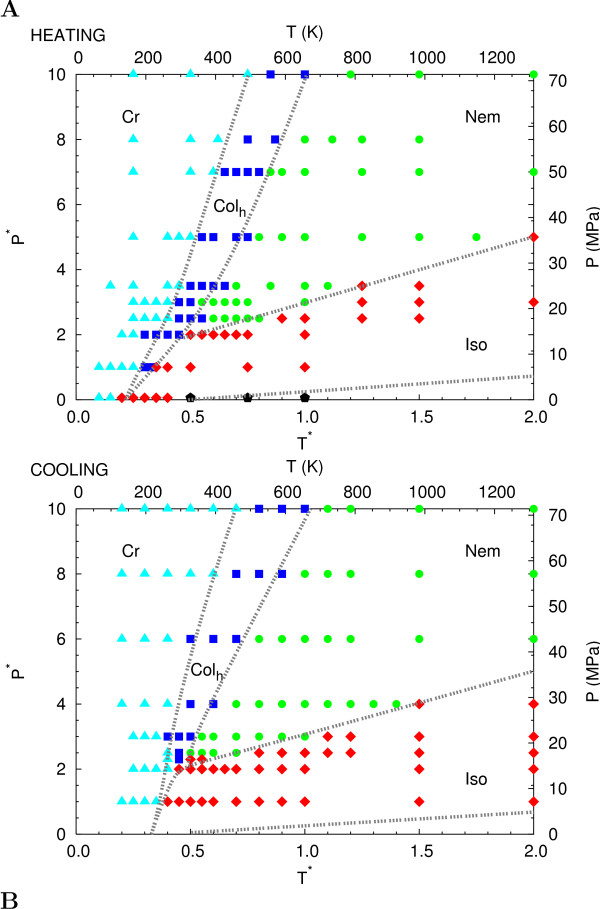
**Heating and cooling P-T phase diagrams of chargeless system**. Pressure-temperature phase diagram of system Q0 derived by heating (A) and cooling (B) sequences of MC simulations. The symbols denote the states where we carried out the simulations: cyano triangles for Cr phase, blue squares for Col_*h*_, green circles for Nem, red diamonds for Iso, black pentagons for Gas. The gray dashed lines are a guide to eyes suggesting the phase boundaries. Both scaled (starred) and real values (see text) for P,T are reported.

The phase diagram presents a rich polymorphism. Upon heating a crystalline (Cr) sample (Figure 2A) at high dimensionless pressures (P* > 2, where P* ≡ Pσ03/ε0
 MathType@MTEF@5@5@+=feaafiart1ev1aaatCvAUfKttLearuWrP9MDH5MBPbIqV92AaeXatLxBI9gBaebbnrfifHhDYfgasaacH8akY=wiFfYdH8Gipec8Eeeu0xXdbba9frFj0=OqFfea0dXdd9vqai=hGuQ8kuc9pgc9s8qqaq=dirpe0xb9q8qiLsFr0=vr0=vr0dc8meaabaqaciaacaGaaeqabaqabeGadaaakeaacqWGqbauiiGacqWFdpWCdaqhaaWcbaGaeGimaadabaGaeG4mamdaaOGaei4la8Iae8xTdu2aaSbaaSqaaiabicdaWaqabaaaaa@355A@), the model exhibits a hexagonal columnar (Col_*h*_) phase and at higher temperatures a nematic (Nem) phase. On the other hand, at low pressures and high temperatures, isotropic liquid (Iso) and gas phases are more stable. In the P* > 2 region the Nem-Col_*h *_and Col_*h*_-Cr transition temperatures increase as pressure increases (positive slope). Also the temperature ranges over which the nematic and the columnar phase are stable, widen with pressure. For P* < 0.5, the columnar phase disappears and the system shows a direct transition from a crystalline to an isotropic, liquid phase. The presence of the nematic phase is typical of rigid GB disks [17-21,24], in which neither the Col_*h*_-Iso transition can be favored by the flexibility of alkyl chains, nor the columnar phase can be stabilized, with respect to the nematic, by microsegregation of cores and chains. Indeed the heating phase diagram looks very similar to the ones reported by Caprion et al. [20] for GB discs with *σ*_*ff*_/*σ*_*ee *_= 0.2, and *ε*_*ee*_/*ε*_*ff *_= 0.1 and 0.2 (while here we have *σ*_*ff*_/*σ*_*ee *_= 0.1948, and *ε*_*ee*_/*ε*_*ff *_= 0.15). We identified the columnar phase as hexagonal with a spatial correlation length of about 15 molecules along the column axis (this was extimated from an additional MC simulation of a larger sample of 8000 molecules), and the crystalline one as interdigitated rectangular.

The sequence of cooling runs (blue dotted line in Figure 2B) shows a phase diagram similar to that obtained from heating runs, but with Cr-Iso and Cr-Col_*h *_equilibrium curves shifted to higher T* values (where T* ≡ *kT*/*ε*_0_) in the P* < 2.5 region and to lower ones for P* > 2.5. Also, the pressure stability range of Iso-Col_*h *_co existence region appears to be strongly reduced with respect to the heating sequence. Indeed, at low pressure the transformation of the nematic phase into a Col_*h *_structure did not proceed along a reversible path and showed significant hysteresis. Relevant hysteresis effects and even monotropic behavior have also been observed experimentally [4].

**Figure 3:**
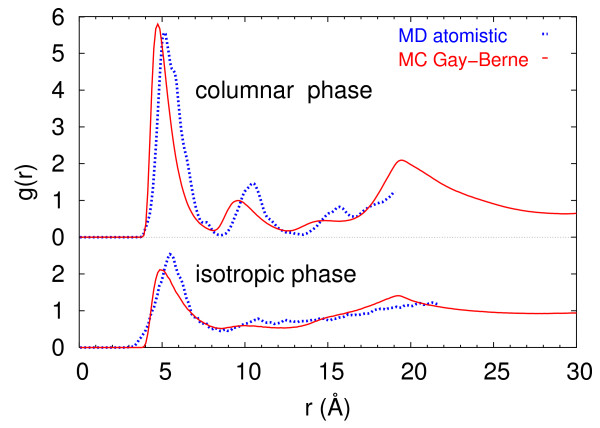
**RDF from atomistic MD and GB MC simulation**. Comparison of the center of mass radial distribution functions calculated from the atomistic MD simulation and the Gay-Berne MC ones both in isotropic and columnar phases. Blue dotted lines for MD data points *T *= 400 (Iso) and *T *= 300 (Col) at *P *= 3 atm; red continuous lines for MC data points T* = 0.4 (Iso) and T* = 0.33 (Col) at P* = 1.0.

**Figure 4:**
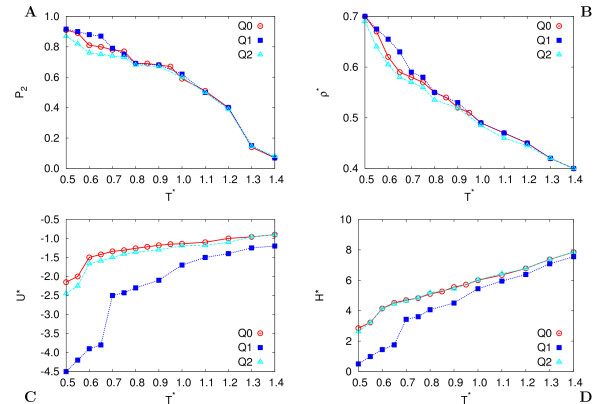
**Observables**. Dependence of the (A) average orientational order parameter *P*_2_, and adimensional (B) number density ρ*, (C) energy U* and (D) enthalpy H* with respect to temperature T*. Results are for GB discs without charges (system Q0, circles), with HT-T charges (system Q1, squares), and with HT-AT charges (system Q2, triangles), at adimensional pressure P* = 3.5.

To compare these structures with those found with the atomistic simulation used to parameterize the GB model, we plotted the radial distribution function from MC simulations of columnar and isotropic phases at dimensionless temperature and pressure equivalent to those of the MD sample (Figure 3). The overall agreement in the shape of these functions, and more specifically in the sequence of maxima and in their height, is satisfactory, even if our coarse grained molecular model exhibits slightly lower intercolumnar distance (broad peak at about 19 Å), when compared with the experimental values for thio and oxa-hexa-alkyl triphenylenes, ranging from about 19 to 25 Å [3,22]. When comparing the experimental and simulated data to match the two temperature ranges, it is customary to scale the simulation energy units in order to overlap both transition temperatures. In this specific case, if we consider the experimental Cr-Col_*h *_transition temperature of 8H-TT (328 K) and compare it to the MC one (T* ≈ 0.5 at P* = 3.5) we obtain an estimate of the dimensioned value of *ε*_0_: ε0d=kTCr−Colhexp/TCr−Colh∗≃1.3
 MathType@MTEF@5@5@+=feaafiart1ev1aaatCvAUfKttLearuWrP9MDH5MBPbIqV92AaeXatLxBI9gBaebbnrfifHhDYfgasaacH8akY=wiFfYdH8Gipec8Eeeu0xXdbba9frFj0=OqFfea0dXdd9vqai=hGuQ8kuc9pgc9s8qqaq=dirpe0xb9q8qiLsFr0=vr0=vr0dc8meaabaqaciaacaGaaeqabaqabeGadaaakeaaiiGacqWF1oqzdaqhaaWcbaGaeGimaadabaGaemizaqgaaOGaeyypa0Jaem4AaSMaemivaq1aa0baaSqaaiabboeadjabbkhaYjabgkHiTiabboeadjabb+gaVjabbYgaSnaaBaaameaacqqGObaAaeqaaaWcbaacbiGae4xzauMae4hEaGNae4hCaahaaOGaei4la8Iaemivaq1aa0baaSqaaiabboeadjabbkhaYjabgkHiTiabboeadjabb+gaVjabbYgaSnaaBaaameaacqqGObaAaeqaaaWcbaGaey4fIOcaaOGaeS4qISJaeGymaeJaeiOla4IaeG4mamdaaa@518E@ kcal/mol. To match the simulation and experiment temperature units, it is then sufficient to rescale it by a factor ε0d
 MathType@MTEF@5@5@+=feaafiart1ev1aaatCvAUfKttLearuWrP9MDH5MBPbIqV92AaeXatLxBI9gBaebbnrfifHhDYfgasaacH8akY=wiFfYdH8Gipec8Eeeu0xXdbba9frFj0=OqFfea0dXdd9vqai=hGuQ8kuc9pgc9s8qqaq=dirpe0xb9q8qiLsFr0=vr0=vr0dc8meaabaqaciaacaGaaeqabaqabeGadaaakeaaiiGacqWF1oqzdaqhaaWcbaGaeGimaadabaGaemizaqgaaaaa@30C6@/*ε*_0_. We opted for this approach, taking for simplicity ε0d
 MathType@MTEF@5@5@+=feaafiart1ev1aaatCvAUfKttLearuWrP9MDH5MBPbIqV92AaeXatLxBI9gBaebbnrfifHhDYfgasaacH8akY=wiFfYdH8Gipec8Eeeu0xXdbba9frFj0=OqFfea0dXdd9vqai=hGuQ8kuc9pgc9s8qqaq=dirpe0xb9q8qiLsFr0=vr0=vr0dc8meaabaqaciaacaGaaeqabaqabeGadaaakeaaiiGacqWF1oqzdaqhaaWcbaGaeGimaadabaGaemizaqgaaaaa@30C6@ = 1 kcal/mol, having also considered that the experimental range of phase transition enthalpies (few kcal/mol in DSC measurements [4]) is well reproduced with this energy scale (see as example the enthalpy values in Figure 4D). Hence, using as unit dimensions *ε*_0 _= 1 kcal/mol and *σ*_0 _= 10 Å considering our definition for T* and P*, we obtained the following scaling factor: *P *= 6.95 P* MPa and *T *= 500 T* K, employed e.g. in Figure 2. Using these data, the limit simulation pressure P* = 2.5 turns out to be 17.5 MPa, and the intermediate P* = 3.5 turn out 24 MPa. While these values are greater than atmospheric pressure, they are not unrealistic, if we compare to the experimental pressures used in [4] for HT-T. Moving to the temperature scale and taking the intermediate pressure P* = 3.5 as reference, the Nem-Col_*h *_transition temperature T* = 0.6 would correspond to a temperature of *T *= 300 K.

### Derivation of electrostatic charges

**Figure 5:**
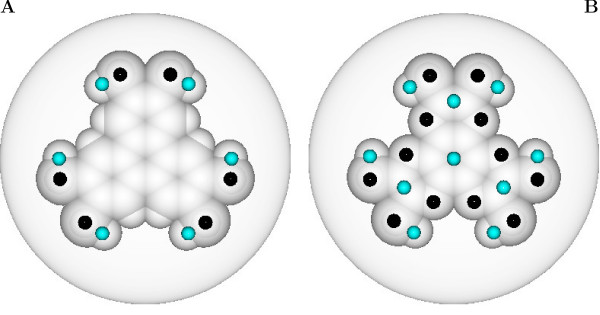
**Electrostatic maps and point charges**. Molecular van der Waals surface and optimal point charges positions represented on a disc with diameter equal to *σ*_*ee *_= 19.25 Å for HT-T (A) and HT-AT (B). Positive charges are shown in light blue, negative in black

We now move to the process of adding to the simple GB model a charge distribution appropriate to the two molecules of interest here. We obtained the electrostatic charges of HT-T and HT-AT, calculated at their equilibrium MP2/6-311G^+ ^geometry, by a direct fit of the molecular electrostatic potential. This procedure naturally determines partial charges for all atoms of the molecule, which could in turn be used to decorate the GB discs previously parametrized. However, exploring the whole phase diagram with such a large number of charges would be unfeasible, because of the computationally overwhelming number of electrostatic interacting pairs to be evaluated. Thus, following a charge-fitting methodology recently developed [23], we derived two minimal sets of point charges that reproduce the main features of both the molecular electrostatic potential and simultaneously the quadrupolar moment. The optimal number of charges suitably placed in the molecular frame resulted in being 12 for HT-T and 22 for HT-AT instead of the original 36 and 30 atomic partial charges (see Figure 5). We see at once from Figure 5 that a major difference of the two charge distributions is the presence of an effective central charge in the case of HT-AT while the core region is essentially neutral for HT-T. As in [23], the quality of the fit has been measured by comparing the intermolecular electrostatic energies of the full and reduced sets for a series of bimolecular configurations, with an overall root mean squared deviation of 0.26 and 0.06 kcal/mol respectively. Regarding the hypothesis that quadrupolar interactions are fundamental in determining the properties of the columnar phase [19], we believe that on the scale of short intermolecular distances, characteristic of the columnar arrangement (<4 Å), and more generally of condensed phases, the central multipolar description is not adequate and a local modelization of electrostatics is needed, justifying the choice of a point charges approach like the present one.

**Figure 6:**
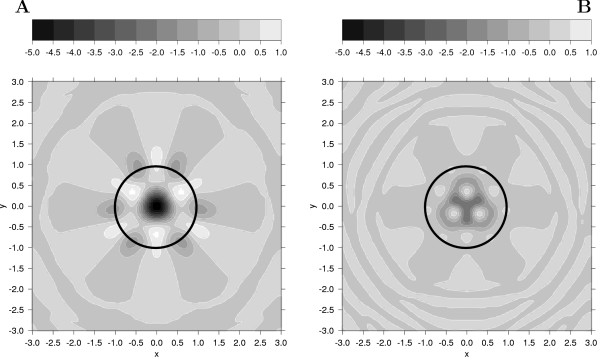
**Contour plots of total intermolecular potential**. Contour plots of the intermolecular potential between two coarse-grained GB particles with embedded charges in face-face arrangement on the z direction at a vertical distance of 0.45 *σ*_0 _and twisted of 60° (A: GB+HT-T charges, B: GB+HT-AT charges). The first particle, whose extension is rendered as a black circle, is kept fixed at the origin, while the second is displaced in the xy plane.

To understand the effect of the charges on the pair interaction energy, it is useful to visualize the electrostatic potential, together with the GB term, for a typical bimolecular configuration occurring in the columnar phase. In Figure 6 we plot such a contour map, calculated for two sliding discs with parallel axes, at a fixed z-distance of 0.45 *σ*_0_, and with varying x and y components of their relative position vector. The twist angle between x molecular axes is fixed to 60°, which gives the maximum interaction [25]. The most striking difference is the presence of a deep minimum in the center of the GB+HT-T map, which, instead, is absent for the GB+HT-AT case, and this accounts for a reduced tendency of the molecules to stack on each other to form a columnar phase.

### Phase behaviour of charged discotics at P* = 3.5

Since the phase diagram of system Q0 gives strong evidence of hysteresis betweeen heating and cooling runs, especially in the low pressure region, as observed experimentally [26], we concentrated our study on the effect of the different charge distributions at an intermediate pressure, P* = 3.5, where the hysteresis effect is relatively negligible, and at temperatures corresponding to isotropic, nematic and columnar phases for system Q0. We therefore performed MC simulations of the GB model with the reduced point charges q∗≡q/(ε0dσ0)1/2
 MathType@MTEF@5@5@+=feaafiart1ev1aaatCvAUfKttLearuWrP9MDH5MBPbIqV92AaeXatLxBI9gBaebbnrfifHhDYfgasaacH8akY=wiFfYdH8Gipec8Eeeu0xXdbba9frFj0=OqFfea0dXdd9vqai=hGuQ8kuc9pgc9s8qqaq=dirpe0xb9q8qiLsFr0=vr0=vr0dc8meaabaqaciaacaGaaeqabaqabeGadaaakeaacqWGXbqCdaahaaWcbeqaaiabgEHiQaaakiabggMi6kabdghaXjabc+caViabcIcaOGGaciab=v7aLnaaDaaaleaacqaIWaamaeaacqWGKbazaaGccqWFdpWCdaWgaaWcbaGaeGimaadabeaakiabcMcaPmaaCaaaleqabaGaeGymaeJaei4la8IaeGOmaidaaaaa@3F04@ representing the HT-T electrostatic potential (system Q1), and that for HT-AT (system Q2).

Figure 4 shows the results for the temperature T* dependence of average orientational order parameter ⟨*P*_2_⟩, number density ⟨ρ*⟩ ≡ σ03
 MathType@MTEF@5@5@+=feaafiart1ev1aaatCvAUfKttLearuWrP9MDH5MBPbIqV92AaeXatLxBI9gBaebbnrfifHhDYfgasaacH8akY=wiFfYdH8Gipec8Eeeu0xXdbba9frFj0=OqFfea0dXdd9vqai=hGuQ8kuc9pgc9s8qqaq=dirpe0xb9q8qiLsFr0=vr0=vr0dc8meaabaqaciaacaGaaeqabaqabeGadaaakeaaiiGacqWFdpWCdaqhaaWcbaGaeGimaadabaGaeG4mamdaaaaa@3085@*N*⟨1/*V*⟩, adimensional energy ⟨U*⟩ ≡ ⟨U*⟩/*ε*_0 _and enthalpy ⟨H*⟩ ≡ ⟨H*⟩/*ε*_0 _per particle for systems Q0, Q1 and Q2. We first notice that all systems show an isotropic and a nematic phase, and that all average observables indicate a similar behavior of the Q1 and Q2 samples in these regions. Conversely the two systems with different charge distributions behave differently at low temperature. The impact of HT-T charges on the total energy is much more important than the one of HT-AT charges, inducing also a slight but significant increase in the orientational order and density at the lowest T* (see also Table 1). This is particularly evident at the Nem-Col_*h *_transition (T* = 0.675), which for the HT-T case occurs at higher temperature than for system Q0, while it is absent in system Q2.

**Table 1:** Observables at P* = 3.5

system	T*	⟨*P*_2_⟩	⟨ρ*⟩	⟨*U*_*tot*_⟩	⟨*U*_*ch*_⟩	phase
Q0	0.50	0.91	0.70	-2.15	-	Col_*h*_
	0.55	0.89	0.67	-2.00	-	Col_*h*_
	0.60	0.81	0.62	-1.50	-	Nem
	0.65	0.80	0.59	-1.42	-	Nem
	0.70	0.78	0.58	-1.34	-	Nem
	0.75	0.77	0.50	-1.31	-	Nem
	0.80	0.69	0.55	-1.26	-	Nem
	0.85	0.69	0.54	-1.22	-	Nem
	0.90	0.68	0.52	-1.18	-	Nem
	0.95	0.67	0.51	-1.15	-	Nem
	1.00	0.59	0.49	-1.14	-	Nem
	1.10	0.51	0.47	-1.10	-	Nem
	1.20	0.4	0.45	-1.00	-	Nem
	1.30	0.14	0.42	-0.96	-	Iso
	1.40	0.07	0.40	-0.90	-	Iso

Q1	0.50	0.92	0.70	-4.50	-2.10	Col_*h*_
	0.55	0.90	0.68	-4.18	-1.98	Col_*h*_
	0.60	0.88	0.66	-3.92	-1.78	Col_*h*_
	0.65	0.87	0.63	-3.83	-1.75	Col_*h*_
	0.70	0.79	0.59	-2.54	-1.00	Nem
	0.75	0.75	0.58	-2.43	-0.90	Nem
	0.80	0.69	0.55	-2.29	-0.90	Nem
	0.90	0.68	0.53	-2.11	-0.80	Nem
	1.00	0.62	0.49	-1.70	-0.55	Nem
	1.10	0.50	0.47	-1.55	-0.42	Nem
	1.20	0.40	0.45	-1.39	-0.38	Nem
	1.30	0.15	0.42	-1.28	-0.29	Iso
	1.40	0.07	0.40	-1.20	-0.27	Iso

Q2	0.50	0.87	0.69	-2.45	-0.28	Nem
	0.55	0.82	0.64	-2.25	-0.20	Nem
	0.60	0.76	0.61	-1.66	-0.14	Nem
	0.65	0.75	0.58	-1.59	-0.13	Nem
	0.70	0.74	0.57	-1.50	-0.12	Nem
	0.75	0.73	0.56	-1.41	-0.11	Nem
	0.80	0.68	0.54	-1.36	-0.10	Nem
	0.90	0.67	0.52	-1.30	-0.10	Nem
	1.00	0.60	0.49	-1.19	-0.09	Nem
	1.10	0.50	0.46	-1.18	-0.09	Nem
	1.20	0.39	0.45	-1.10	-0.09	Nem
	1.30	0.15	0.42	-1.00	-0.09	Iso
	1.40	0.08	0.40	-0.90	-0.08	Iso

Average simulation values for second rank order parameter ⟨*P*_2_⟩, number density ⟨ρ*⟩ dimensionless total energy 〈Utot∗〉
 MathType@MTEF@5@5@+=feaafiart1ev1aaatCvAUfKttLearuWrP9MDH5MBPbIqV92AaeXatLxBI9gBaebbnrfifHhDYfgasaacH8akY=wiFfYdH8Gipec8Eeeu0xXdbba9frFj0=OqFfea0dXdd9vqai=hGuQ8kuc9pgc9s8qqaq=dirpe0xb9q8qiLsFr0=vr0=vr0dc8meaabaqaciaacaGaaeqabaqabeGadaaakeaacqGHPms4cqWGvbqvdaqhaaWcbaGaeeiDaqNaee4Ba8MaeeiDaqhabaGaey4fIOcaaOGaeyOkJepaaa@36CB@ and electostatic energy 〈Uch∗〉
 MathType@MTEF@5@5@+=feaafiart1ev1aaatCvAUfKttLearuWrP9MDH5MBPbIqV92AaeXatLxBI9gBaebbnrfifHhDYfgasaacH8akY=wiFfYdH8Gipec8Eeeu0xXdbba9frFj0=OqFfea0dXdd9vqai=hGuQ8kuc9pgc9s8qqaq=dirpe0xb9q8qiLsFr0=vr0=vr0dc8meaabaqaciaacaGaaeqabaqabeGadaaakeaacqGHPms4cqWGvbqvdaqhaaWcbaGaee4yamMaeeiAaGgabaGaey4fIOcaaOGaeyOkJepaaa@352C@, for discs models Q0,Q1, Q2 at P* = 3.5. Statistical averages are ± 0.01 for all quantities.

**Figure 7:**
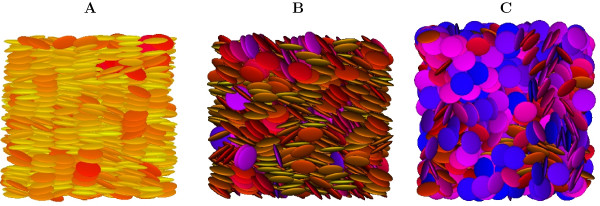
**HT-T, MC Snapshots**. Snapshots from MC simulations of system Q1 at T* = 0.6 (A), T* = 0.8 (B), T* = 1.4 (C). The orientation of the molecules, with respect to the director, is rendered using a color coding ranging from yellow (parallel) to blue (perpendicular).

**Figure 8:**
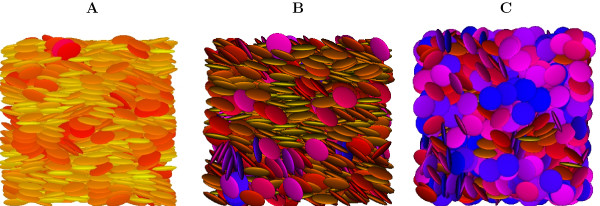
**HT-AT, MC Snapshots**. Snapshots from MC simulations of system Q2 at T* = 0.6 (A), T* = 0.8 (B), T* = 1.4 (C). The orientation of the molecules, with respect to the director, is rendered using a color coding ranging from yellow (parallel) to blue (perpendicular).

**Figure 9:**
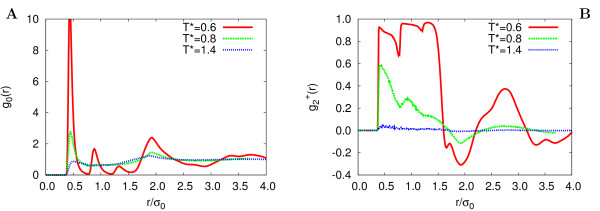
**HT-T, Correlation functions**. Radial correlation function *g*_0_(*r*) (A) and second rank anisotropy g2+(r)
 MathType@MTEF@5@5@+=feaafiart1ev1aaatCvAUfKttLearuWrP9MDH5MBPbIqV92AaeXatLxBI9gBaebbnrfifHhDYfgasaacH8akY=wiFfYdH8Gipec8Eeeu0xXdbba9frFj0=OqFfea0dXdd9vqai=hGuQ8kuc9pgc9s8qqaq=dirpe0xb9q8qiLsFr0=vr0=vr0dc8meaabaqaciaacaGaaeqabaqabeGadaaakeaacqWGNbWzdaqhaaWcbaGaeGOmaidabaGaey4kaScaaOGaeiikaGIaemOCaiNaeiykaKcaaa@332D@ (B) from MC simulations of system Q1 at T* = 0.6 (red continuous line), 0.8 (green dotted line), 1.4 (blue dashed line).

**Figure 10:**
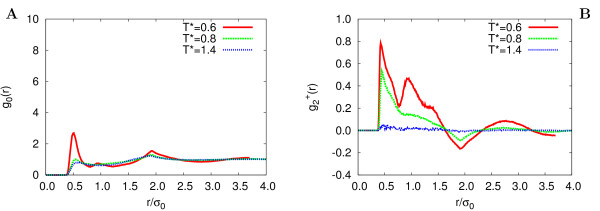
**HT-AT, Correlation functions**. Radial correlation function *g*_0_(*r*) (A) and second rank anisotropy g2+(r)
 MathType@MTEF@5@5@+=feaafiart1ev1aaatCvAUfKttLearuWrP9MDH5MBPbIqV92AaeXatLxBI9gBaebbnrfifHhDYfgasaacH8akY=wiFfYdH8Gipec8Eeeu0xXdbba9frFj0=OqFfea0dXdd9vqai=hGuQ8kuc9pgc9s8qqaq=dirpe0xb9q8qiLsFr0=vr0=vr0dc8meaabaqaciaacaGaaeqabaqabeGadaaakeaacqWGNbWzdaqhaaWcbaGaeGOmaidabaGaey4kaScaaOGaeiikaGIaemOCaiNaeiykaKcaaa@332D@ (B) from MC simulations of system Q2 at T* = 0.6 (red continuous line), 0.8 (green dotted line), 1.4 (blue dashed line).

The different phase behaviour between systems Q1 and Q2 is also apparent from the snapshots shown in Figures 7, 8, where the molecular orientation with respect to the director is rendered using a color map ranging from yellow (parallel) to blue (perpendicular). We can see that clustering of particles is comparable for both systems at nematic (T* = 0.8) and isotropic (T* = 1.4) temperatures, while for the lowest temperature (T* = 0.6) of the HT-T discs (system Q1), columns are well defined and extend across the whole sample. On the contrary, even at this temperature system Q2 appears to be nematic. A more quantitative structural characterization of the phases is achieved by monitoring the positional order through the calculation of the radial distribution function g_0_(*r*) and its second rank anisotropy g2+(r)
 MathType@MTEF@5@5@+=feaafiart1ev1aaatCvAUfKttLearuWrP9MDH5MBPbIqV92AaeXatLxBI9gBaebbnrfifHhDYfgasaacH8akY=wiFfYdH8Gipec8Eeeu0xXdbba9frFj0=OqFfea0dXdd9vqai=hGuQ8kuc9pgc9s8qqaq=dirpe0xb9q8qiLsFr0=vr0=vr0dc8meaabaqaciaacaGaaeqabaqabeGadaaakeaacqWGNbWzdaqhaaWcbaGaeGOmaidabaGaey4kaScaaOGaeiikaGIaemOCaiNaeiykaKcaaa@332D@. The former gives the probability of finding a molecule at a distance *r *from the one at the origin, relative to the probability expected for a completely uniform distribution at the same density, while g2+(r)
 MathType@MTEF@5@5@+=feaafiart1ev1aaatCvAUfKttLearuWrP9MDH5MBPbIqV92AaeXatLxBI9gBaebbnrfifHhDYfgasaacH8akY=wiFfYdH8Gipec8Eeeu0xXdbba9frFj0=OqFfea0dXdd9vqai=hGuQ8kuc9pgc9s8qqaq=dirpe0xb9q8qiLsFr0=vr0=vr0dc8meaabaqaciaacaGaaeqabaqabeGadaaakeaacqWGNbWzdaqhaaWcbaGaeGOmaidabaGaey4kaScaaOGaeiikaGIaemOCaiNaeiykaKcaaa@332D@ = *V*/(4*πr*^2^*N*)⟨*δ*(*r *- *r*_*ij*_)*P*_2 _(cos *β*_*ij*_)⟩_*ij *_[27], where *β*_*ij *_is the angle between the intermolecular vector and the phase director, is a measure of the tendency of molecules to align in the face-face arrangement. In Figures 9A, 10A, we show the system Q1 and Q2 pair distribution functions for temperatures T* = 0.6, 0.8, 1.4. At the lowest temperature g_0_(*r*) is very structured, indicating a high degree of positional order. The first three peaks at *r *≈ 0.4, 0.8, 1.2 *σ*_0 _correspond to neighbouring molecules stacked in the same column (face-face). The fourth peak is due to molecules (edge-edge) belonging to neighbouring columns (see also Figure 7). Less structured features are found in *g*_0_(*r*) at T* = 0.6 of system Q2, while at T* = 0.8, 1.4 the radial correlation functions show no positional ordering for both systems in study: edge-edge configurations are approximately as frequent as face-face. Consistently with the onset of the columnar phase the second rank anisotropy g2+(r)
 MathType@MTEF@5@5@+=feaafiart1ev1aaatCvAUfKttLearuWrP9MDH5MBPbIqV92AaeXatLxBI9gBaebbnrfifHhDYfgasaacH8akY=wiFfYdH8Gipec8Eeeu0xXdbba9frFj0=OqFfea0dXdd9vqai=hGuQ8kuc9pgc9s8qqaq=dirpe0xb9q8qiLsFr0=vr0=vr0dc8meaabaqaciaacaGaaeqabaqabeGadaaakeaacqWGNbWzdaqhaaWcbaGaeGOmaidabaGaey4kaScaaOGaeiikaGIaemOCaiNaeiykaKcaaa@332D@ (in Figures 9B, 10B) exhibits a rich structure even at large molecular separations: a close look at the curves shows three positive maxima for the intra-column neighbouring pairs (intermolecular vector parallel to the director) and a fourth negative peak corresponding to edge-edge molecules belonging to adjacent columns (intermolecular vector perpendicular to the director).

Also in the isotropic phase (T* = 1.4) the HT-T system shows evidences of short range spatial and orientational correlation, i.e. also at high temperature we find couples of molecules in face-face arrangement. This configuration, like observed precedently for the contour plots, is favored by the HT-T charge distribution and strengthens the interaction between molecules at all temperatures (see U* in Figure 4), with particular stabilization of the columnar phase.

## Conclusion

We derived a new parameterization of the GB potential by fitting the total LJ pair intermolecular potential from an atomistic MD simulation of hexa-alkyl-thio triphenylene. MC simulations show that the resulting molecular model exhibits isotropic, columnar and crystalline phases at low pressures, while at higher pressures (P* > 2) a wide nematic phase appears and the columnar phase turns out enlarged. We believe this parametrization can be successfully employed as the basis for molecular level simulations of triphenylene molecules; notably different core sizes can be modelled by minor modifications of *σ*_*ee *_and *σ*_*ff *_parameters.

Adding the HT-T and HT-AT charges to the GB model at the selected pressure P* = 3.5, we found that the intermolecular electrostatic potential among the cores is fundamental in stabilizing/destabilizing columnar phases; in particular the HT-T charge distribution stabilizes the columnar structure, while the HT-AT distribution suppresses its formation in favor of the nematic phase. This study therefore suggests that hexa-alkyl-thio azatriphenylenes do not form columnar phases due to the unfavorable charge distribution of the azatriphenylene core.

## Experimental

### MD simulation details

We ran atomistic MD simulations on systems of N = 40 8HT-T molecules, initially stacked in four columns of ten molecules each. We employed the AMBER-OPLS force field [28,29] and we set all the point charges to zero, since the core-core electrostatic interaction have been taken into account in a separate specific model and the alkyl chains are approximately neutral.

On heating at ambient pressure, the 8HT-T solid compound melts from a crystalline phase to a columnar hexagonal phase at 328 K, and to the isotropic phase at 360 K [30]; in the simulation we used a temperature of 300 K and at a pressure of 3 atm to compensate the absence of electrostatic interactions and while remaining in a region of stability of the columnar arrangement. The simulations were run in the isothermal-isobaric ensemble with the Orac code [31]:, velocity scaling thermostat and isotropic Parrinello-Rahman barostat [32]: after an equilibration period of about 2 ns, we accumulated averages and trajectories for 2 ns more.

### MC simulation details

Systems Q0, Q1, Q2 were studied from MC cooling and heating scans at constant pressure, with temperature steps of ΔT* ≤ 0.1. The equilibration stage of each simulation consisted of 300–400 thousand cycles and was followed by a production stage of 100 thousand cycles. Each MC cycle consists of trial displacements and reorientations of all the molecules and, with 10% probability, of a trial box side change. The simulation box was orthogonal with 3D periodic boundary conditions.

In systems with charges (systems Q1 and Q2) the long range electrostatic interactions were computed using the method proposed by Wolf *et al*. in [33], which is computationally more efficient than traditional Ewald summations. For the systems considered the compromise between accuracy and speed, with respect to standard Ewald method, is good provided that the truncation radius and the damping parameter *α *are adequately chosen (in this case *α *= 8). However, because of the high number of charges considered, simulation on systems Q1 and Q2 were run on pools of 8 processor using an MPI fortran77 program and employing a data replicated approach to distribute the energy computation between the threads.

In all experiments we considered a number of particles *N *= 1000, reduced pressures P* ranging from 0.01 to 10, and a cutoff *r*_*c *_of 3.5 *σ*_0_, where the energy unit *ε*_0 _has initially been set to 30 kcal/mol and the distance unit *σ*_0 _to 10 Å. Phase transitions were located from the variation of the radial distribution function, of the second rank orientational order parameter ⟨*P*_2_⟩ = 〈32(zi⋅Z)2−12〉
 MathType@MTEF@5@5@+=feaafiart1ev1aaatCvAUfKttLearuWrP9MDH5MBPbIqV92AaeXatLxBI9gBaebbnrfifHhDYfgasaacH8akY=wiFfYdH8Gipec8Eeeu0xXdbba9frFj0=OqFfea0dXdd9vqai=hGuQ8kuc9pgc9s8qqaq=dirpe0xb9q8qiLsFr0=vr0=vr0dc8meaabaqaciaacaGaaeqabaqabeGadaaakeaadaaadeqaamaalaaabaGaeG4mamdabaGaeGOmaidaamaabmaabaacbeGae8NEaO3aaSbaaSqaaiab=LgaPbqabaGccqGHflY1cqWFAbGwaiaawIcacaGLPaaadaahaaWcbeqaaiab=jdaYaaakiabgkHiTmaalaaabaGae8xmaedabaGae8NmaidaaaGaayzkJiaawQYiaaaa@3C7C@ and from the often small discontinuities in the enthalpy H* = U* + P*/ρ*.
